# Supplementary material for: Detection and tracking of barchan dunes using artificial intelligence
Source: Sci Rep. 2024 Aug 8;14:18381. doi: 10.1038/s41598-024-67893-y (PMC11306226; doi:10.1038/s41598-024-67893-y)
Supplement: Supplementary file 1 — Supplementary Information. [file 41598_2024_67893_MOESM1_ESM.zip › legend_suppl_videos.pdf]

Legends of supplementary videos

Chasing.mp4 – movie showing the automatic detection of two subaqueous barchans migrating without touching each other (chasing process).

Exchange.mp4 – movie showing the automatic detection of one subaqueous barchan that collides with another one, with the subsequent ejection of a new (third) barchan dune (exchange process).

Fragmentation_1.mp4 – movie showing the automatic detection of two subaqueous barchans migrating without touching each other, with the split of the downstream barchan into two during the process (fragmentation process).

Fragmentation_2.mp4 – movie showing the automatic detection of two subaqueous barchans migrating without touching each other, with the split of the downstream barchan into two during the process (fragmentation process).
