# Supplementary material for: Detection and tracking of barchan dunes using artificial intelligence
Source: Sci Rep. 2024 Aug 8;14:18381. doi: 10.1038/s41598-024-67893-y (PMC11306226; doi:10.1038/s41598-024-67893-y)
Supplement: Supplementary file 1 — Supplementary Information. [file 41598_2024_67893_MOESM1_ESM.zip › supplementary_information_pdf.pdf]

Supplementary Information: Detection and tracking of barchan dunes using Artificial Intelligence

Esteban A. Cúñez and Erick M. Franklin[a)]

Faculdade de Engenharia Mecânica, Universidade Estadual de Campinas (UNICAMP), Rua Mendeleyev, 200, Campinas, SP, Brazil

(Dated: 3 June 2024)

[a)]Electronic mail: erick.franklin@unicamp.br; Corresponding author

Introduction

This supplementary information presents additional graphics and movies showing examples of the tracking of bedforms along movie frames and in satellite images. We note that data supporting our findings are available in open repositories[1–6].

Legends of supplementary videos

`Chasing.mp4` – movie showing the automatic detection of two subaqueous barchans migrating without touching each other (chasing process).

`Exchange.mp4` – movie showing the automatic detection of one subaqueous barchan that collides with another one, with the subsequent ejection of a new (third) barchan dune (exchange process).

`Fragmentation_1.mp4` – movie showing the automatic detection of two subaqueous barchans migrating without touching each other, with the split of the downstream barchan into two during the process (fragmentation process).

`Fragmentation_2.mp4` – movie showing the automatic detection of two subaqueous barchans migrating without touching each other, with the split of the downstream barchan into two during the process (fragmentation process).

Additional figures

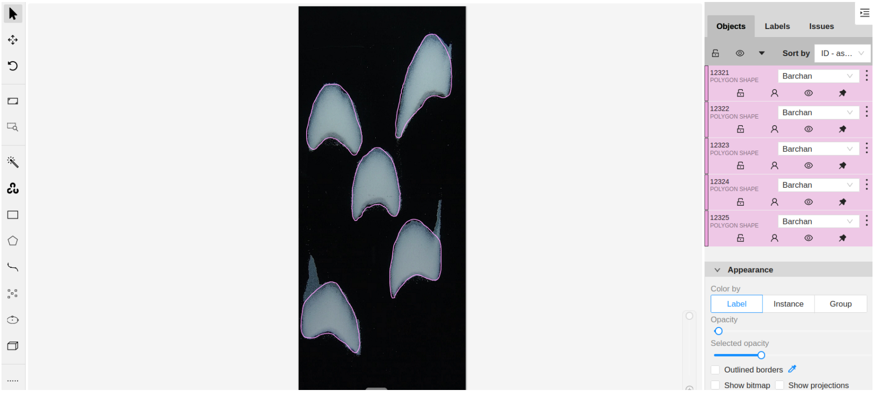

FIG. 1. Example of dune labeling using the CVAT platform (https://www.cvat.ai/). Case for subaqueous experiments.

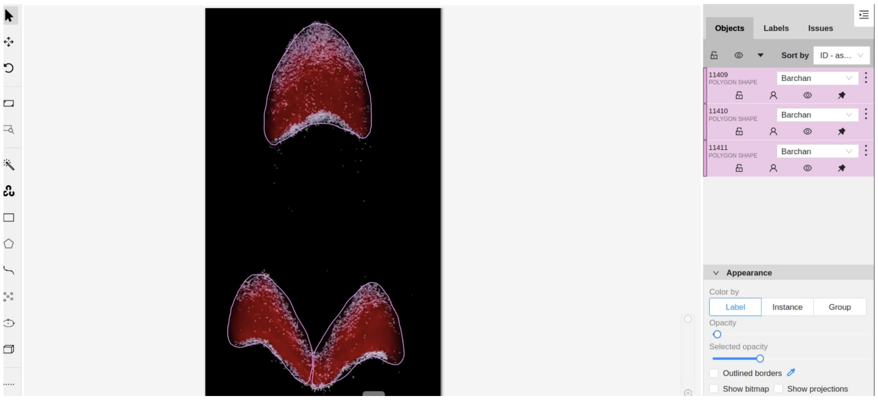

FIG. 2. Example of dune labeling using the CVAT platform (https://www.cvat.ai/). Case for subaqueous experiments.

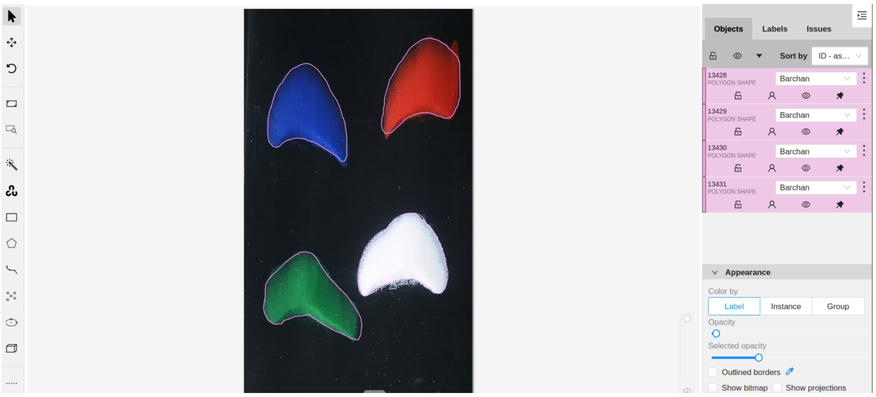

FIG. 3. Example of dune labeling using the CVAT platform (https://www.cvat.ai/). Case for subaqueous experiments.

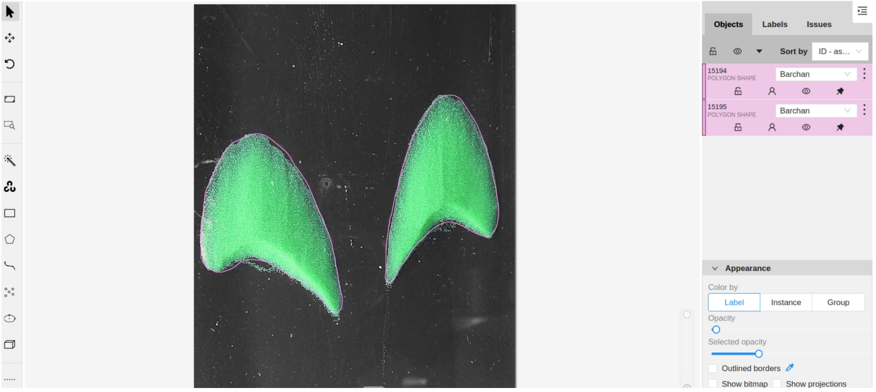

FIG. 4. Example of dune labeling using the CVAT platform (https://www.cvat.ai/). Case for subaqueous experiments.

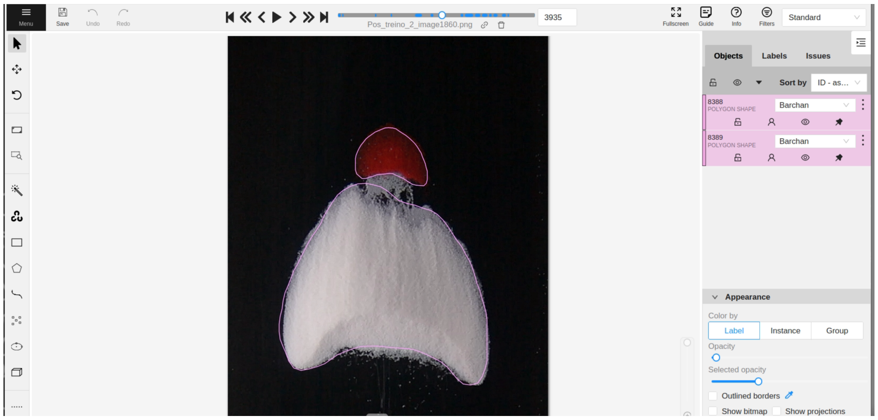

FIG. 5. Example of dune labeling using the CVAT platform (https://www.cvat.ai/). Case for subaqueous experiments in the initial stage of an exchange pattern.

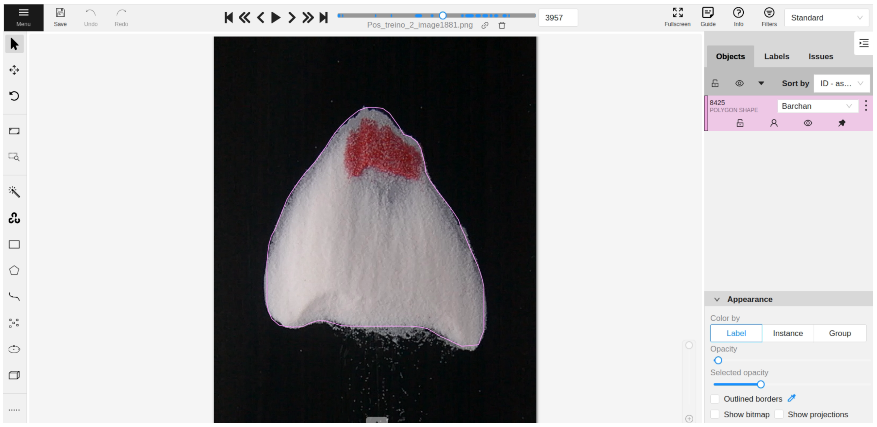

FIG. 6. Example of dune labeling using the CVAT platform (https://www.cvat.ai/). Case for subaqueous experiments in the intermediate stage of an exchange pattern.

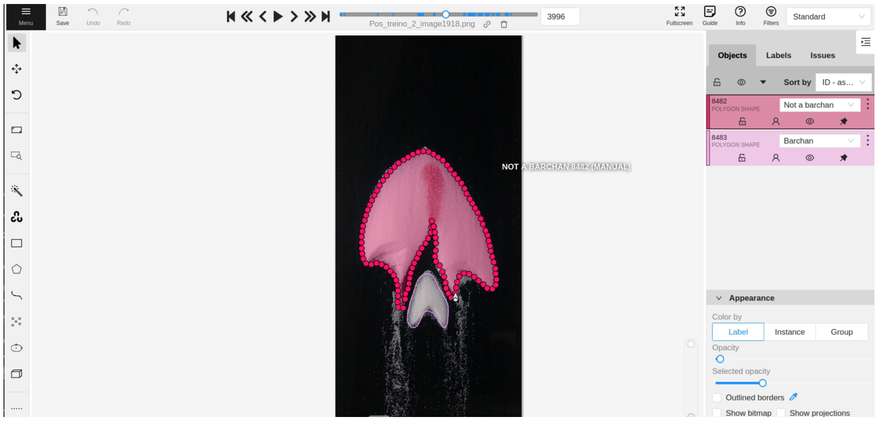

FIG. 7. Example of dune labeling using the CVAT platform (https://www.cvat.ai/). Case for subaqueous experiments in the final stage of an exchange pattern.

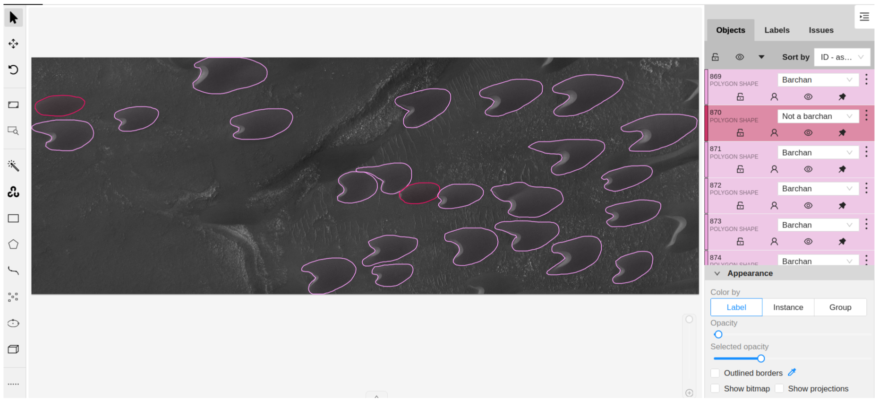

FIG. 8. Example of dune labeling using the CVAT platform (https://www.cvat.ai/). Case for Martian dunes. Courtesy NASA/JPL-Caltech/UArizona.

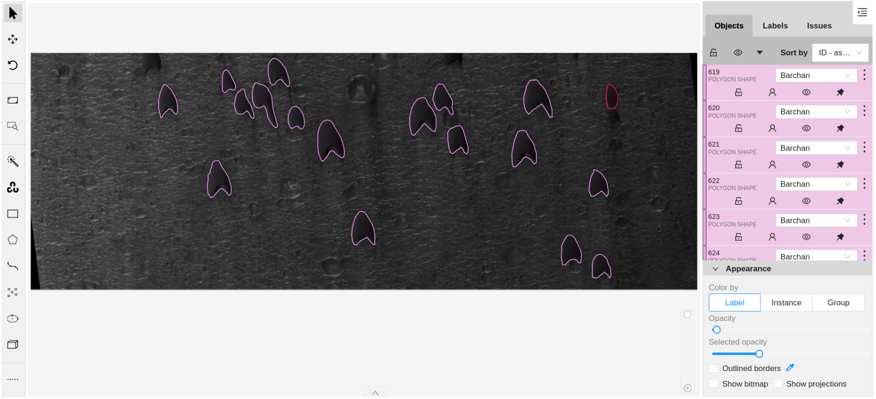

FIG. 9. Example of dune labeling using the CVAT platform (https://www.cvat.ai/). Case for Martian dunes Courtesy NASA/JPL-Caltech/UArizona.

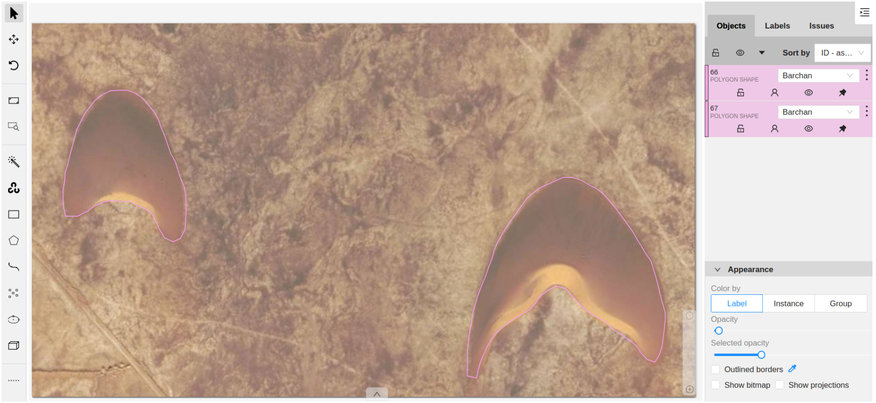

FIG. 10. Example of dune labeling using the CVAT platform (https://www.cvat.ai/). Case for eolian (Earth's) dunes. Courtesy Google Earth.

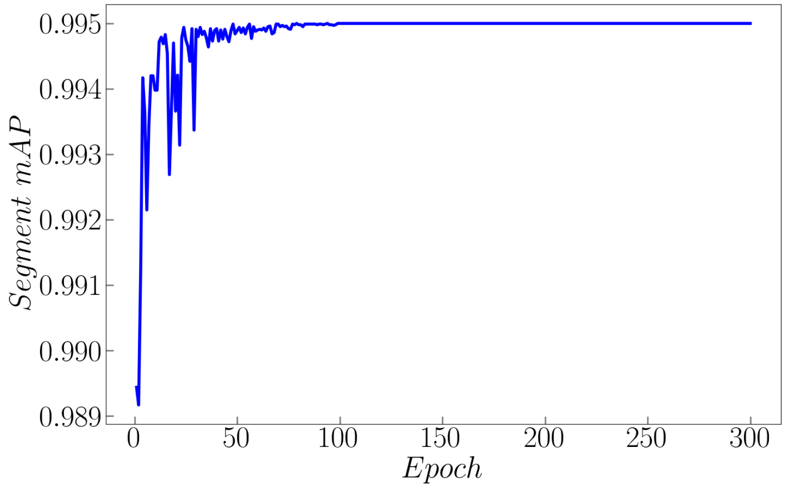

FIG. 11. Evolution of the mean average precision *mAP* along the epochs for images from experiments. For this training we used 7455 images, of which 876 for validation. The used images are available in an open repository[5].

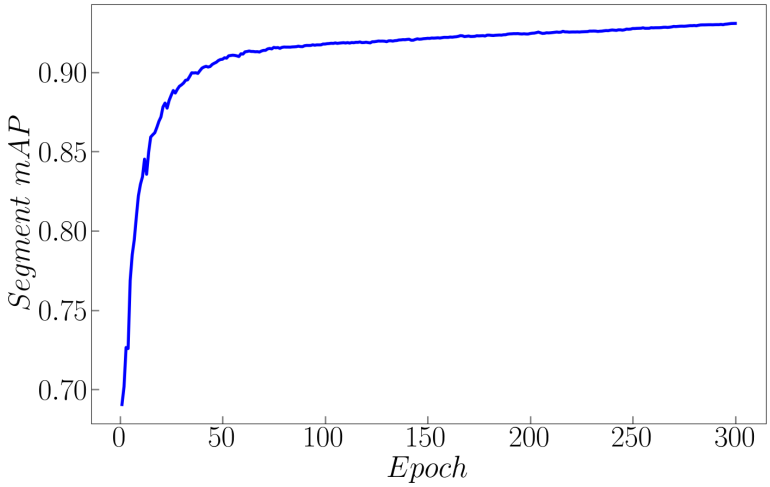

FIG. 12. Evolution of the mean average precision *mAP* along the epochs for satellite images. For this training we used 12395 images, and 2376 for validation. The used images are available in an open repository[6].

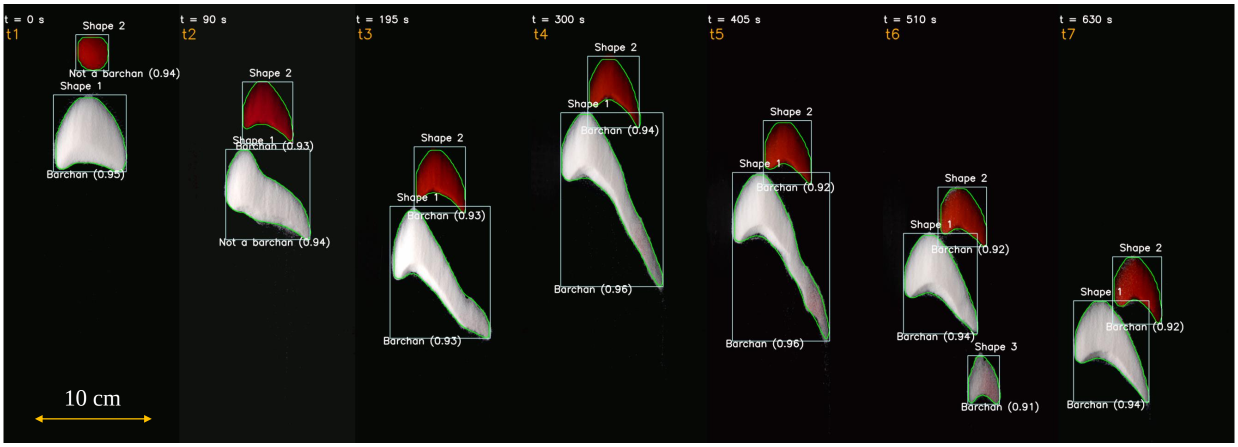

FIG. 13. Snapshots placed side by side of two barchans interacting in a pattern called *fragmentation*[7]. The images were taken from the open repository[1] created by Assis and Franklin[7], from which we selected some time instants shown on the top of each snapshot. The instants were numbered from $t1$ to $t7$ (shown in orange on the top), a length scale is shown on the bottom left, the boxes enclosing the identified objects are shown in white, and the outline of objects are shown in green. Each object has a label (Shape 1 to Shape 3) which is kept until the last image, and the classes *Barchan* and *Not a barchan* are shown for each object with the corresponding confidence score. In the images, water flow is from top to bottom.

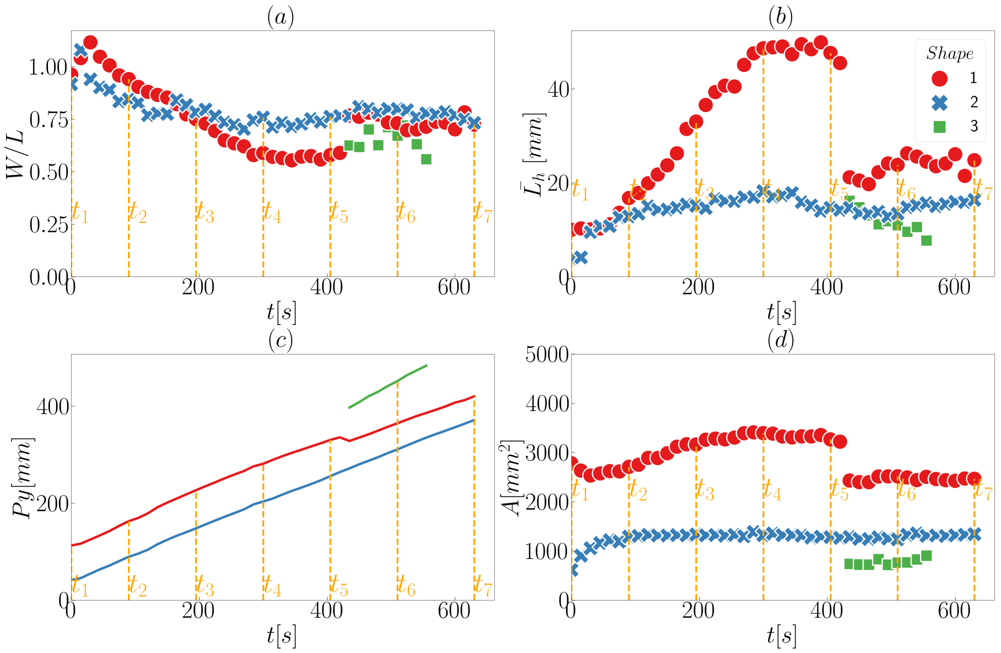

FIG. 14. Time evolution of morphology and position of interacting bedforms for the case shown in Fig. 13: (a) aspect ratio W/L; (b) mean horn length $\overline{L}_h$; (c) longitudinal position P_y of the centroid of bedforms; and (d) Area A of the horizontal projection of the bedform surface. Time instants corresponding to the snapshots of Fig. 13 are shown in all panels. The mean horn length is $\overline{L}_h = (L_{hr} + L_{hl})/2$.

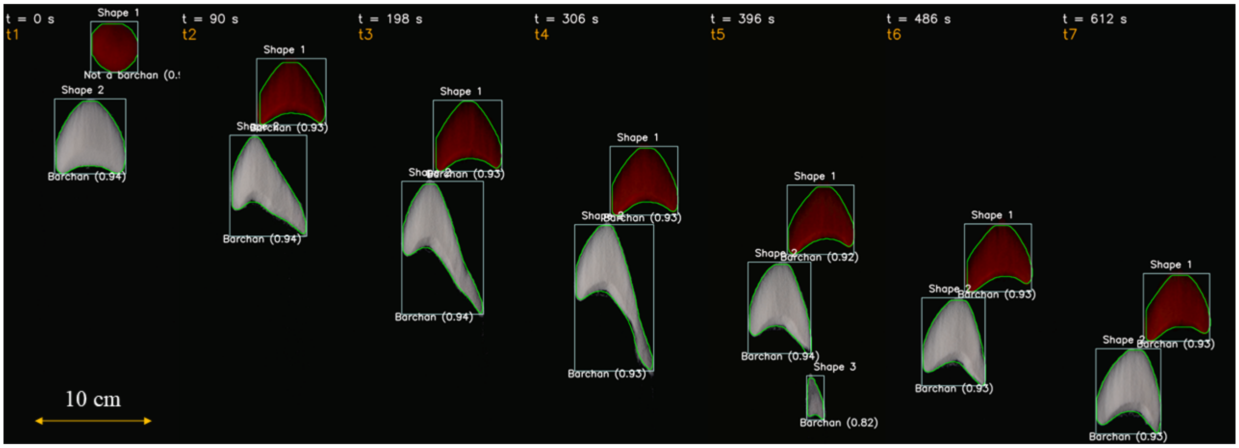

FIG. 15. Snapshots placed side by side of of two barchans interacting in a pattern called *fragmentation*[7]. The images were taken from the open repository[1] created by Assis and Franklin[7], from which we selected some time instants shown on the top of each snapshot. The instants were numbered from $t1$ to $t7$ (shown in orange on the top), a length scale is shown on the bottom left, the boxes enclosing the identified objects are shown in white, and the outline of objects are shown in green. Each object has a label (Shape 1 to Shape 3) which is kept until the last image, and the classes *Barchan* and *Not a barchan* are shown for each object with the corresponding confidence score. In the images, water flow is from top to bottom.

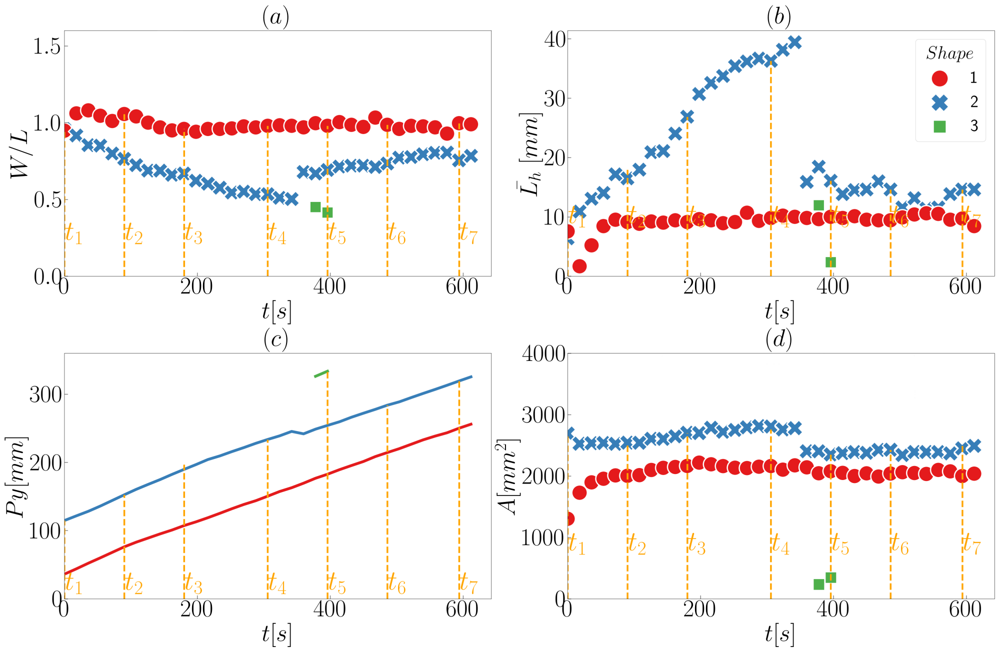

FIG. 16. Time evolution of morphology and position of interacting bedforms for the case shown in Fig. 15: (a) aspect ratio W/L; (b) mean horn length $\overline{L}_h$; (c) longitudinal position P_y of the centroid of bedforms; and (d) Area A of the horizontal projection of the bedform surface. Time instants corresponding to the snapshots of Fig. 15 are shown in all panels. The mean horn length is $\overline{L}_h = (L_{hr} + L_{hl})/2$.

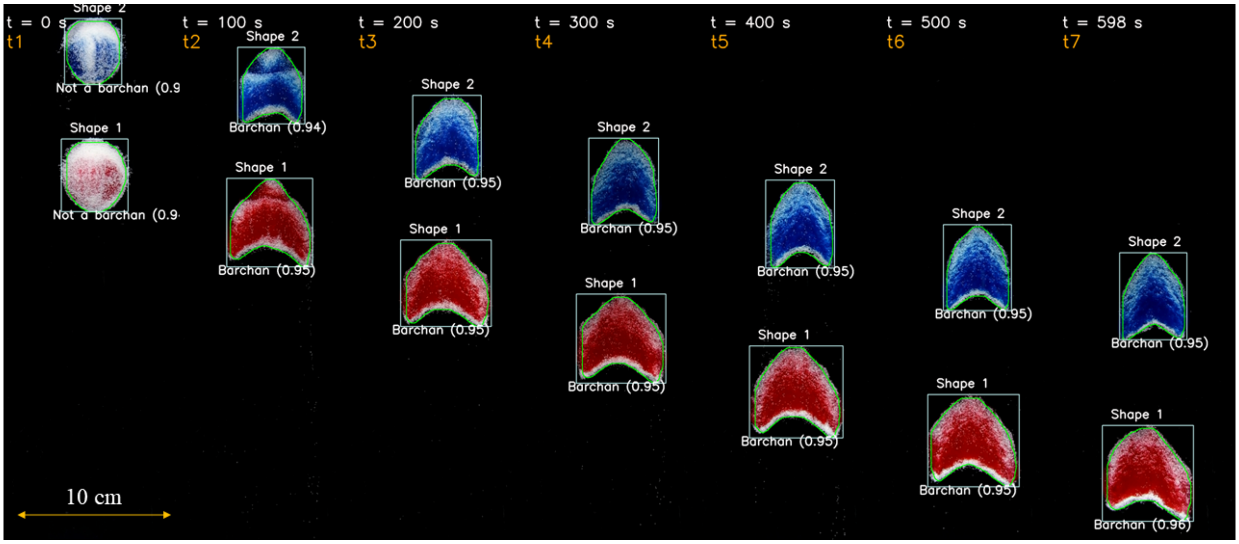

FIG. 17. Snapshots placed side by side of of two barchans interacting in a pattern called *chasing*[7]. The images were taken from the open repository[1] created by Assis and Franklin[7], from which we selected some time instants shown on the top of each snapshot. The instants were numbered from $t1$ to $t7$ (shown in orange on the top), a length scale is shown on the bottom left, the boxes enclosing the identified objects are shown in white, and the outline of objects are shown in green. Each object has a label (Shape 1 and Shape 2) which is kept until the last image, and the classes *Barchan* and *Not a barchan* are shown for each object with the corresponding confidence score. In the images, water flow is from top to bottom.

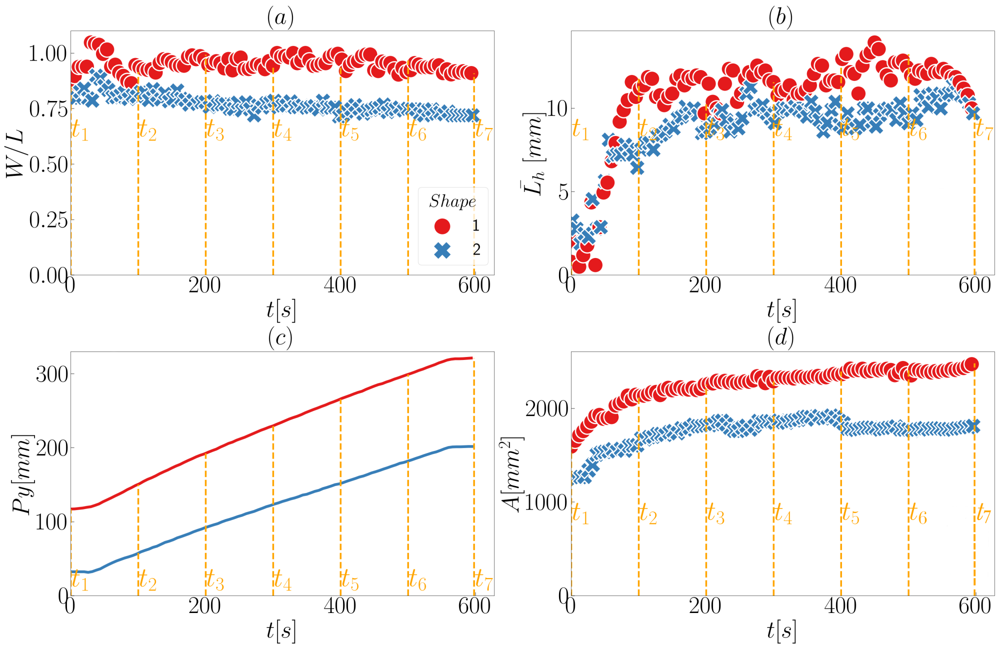

FIG. 18. Time evolution of morphology and position of interacting bedforms for the case shown in Fig. 17: (a) aspect ratio W/L; (b) mean horn length $\overline{L}_h$; (c) longitudinal position P_y of the centroid of bedforms; and (d) Area A of the horizontal projection of the bedform surface. Time instants corresponding to the snapshots of Fig. 17 are shown in all panels. The mean horn length is $\overline{L}_h = (L_{hr} + L_{hl})/2$.

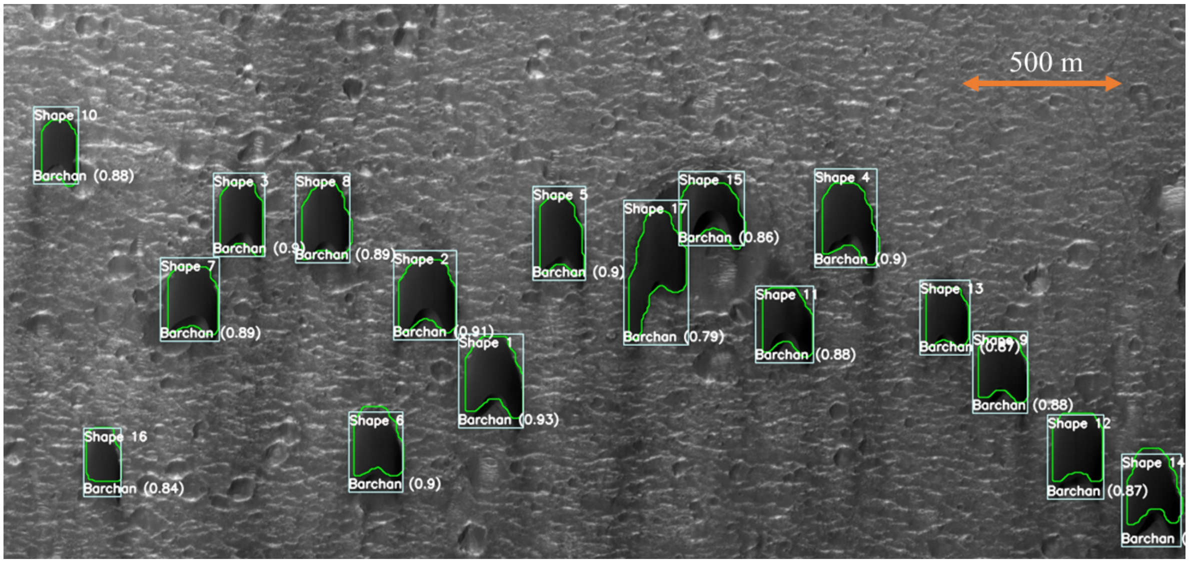

FIG. 19. HiRISE image[8] showing a field of barchans undergoing complex interactions on the surface of Mars: 23.190° latitude (centered), 339.585° longitude (East), spacecraft altitude 287.3 km. Courtesy NASA/JPL-Caltech/UArizona. The detection boxes, class type, confidence score and outline are superposed with the image.

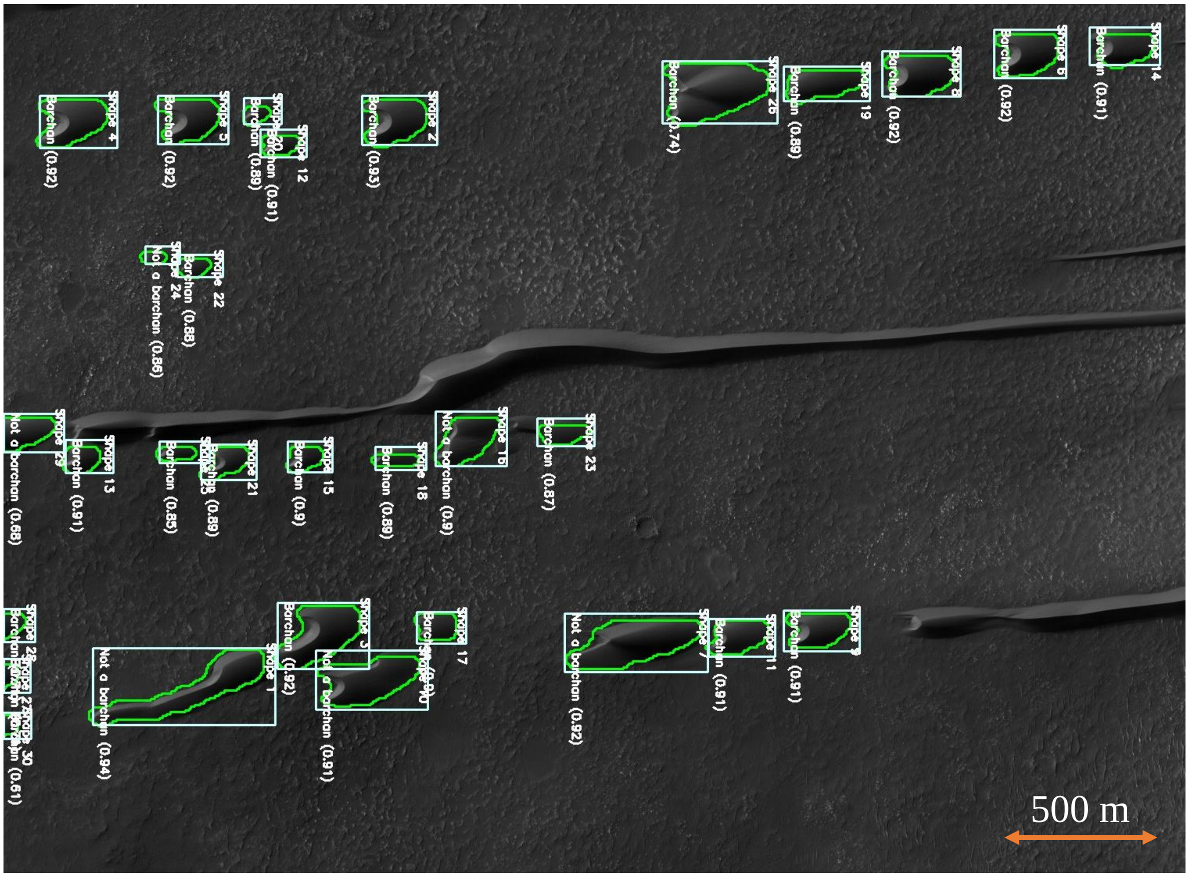

FIG. 20. HiRISE image[8] showing a field of barchans undergoing complex interactions on the surface of Mars: -41.488° latitude (centered), 44.589° longitude (East), spacecraft altitude 253.8 km. Courtesy NASA/JPL-Caltech/UArizona. The detection boxes, class type, confidence score and outline are superposed with the image.

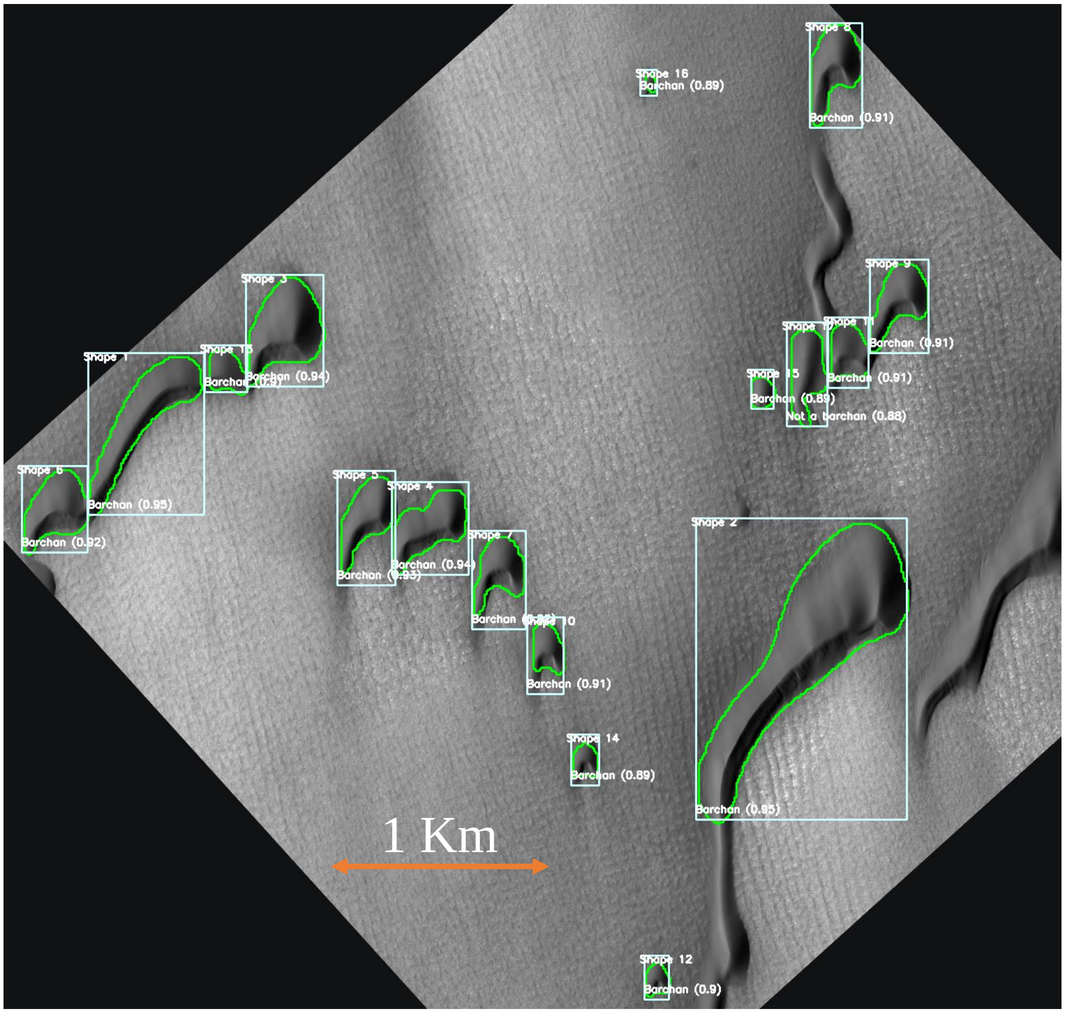

FIG. 21. HiRISE image[8] showing a field of barchans undergoing complex interactions on the surface of Mars: 74.884° latitude (centered), 279.346° longitude (East), spacecraft altitude 317.6 km. Courtesy NASA/JPL-Caltech/UArizona. The detection boxes, class type, confidence score and outline are superposed with the image.

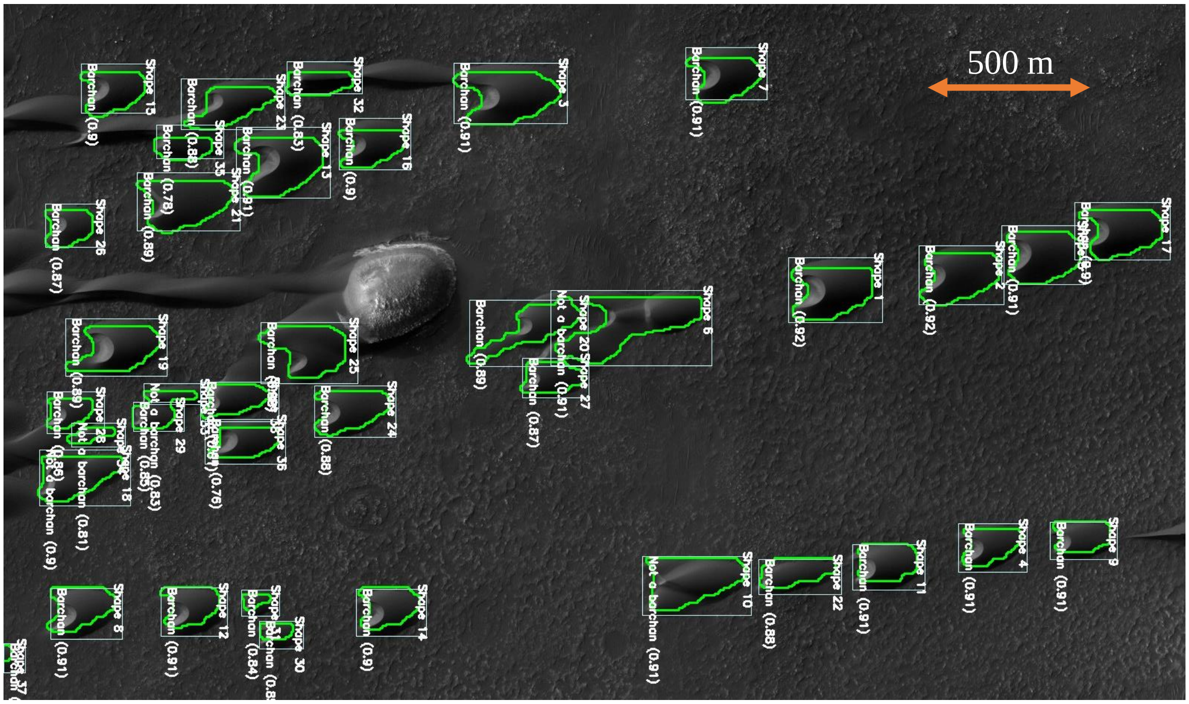

FIG. 22. HiRISE image[8] showing a field of barchans undergoing complex interactions on the surface of Mars: -41.488° latitude (centered), 44.589° longitude (East), spacecraft altitude 253.8 km. Courtesy NASA/JPL-Caltech/UArizona. The detection boxes, class type, confidence score, and outline are superposed with the image.

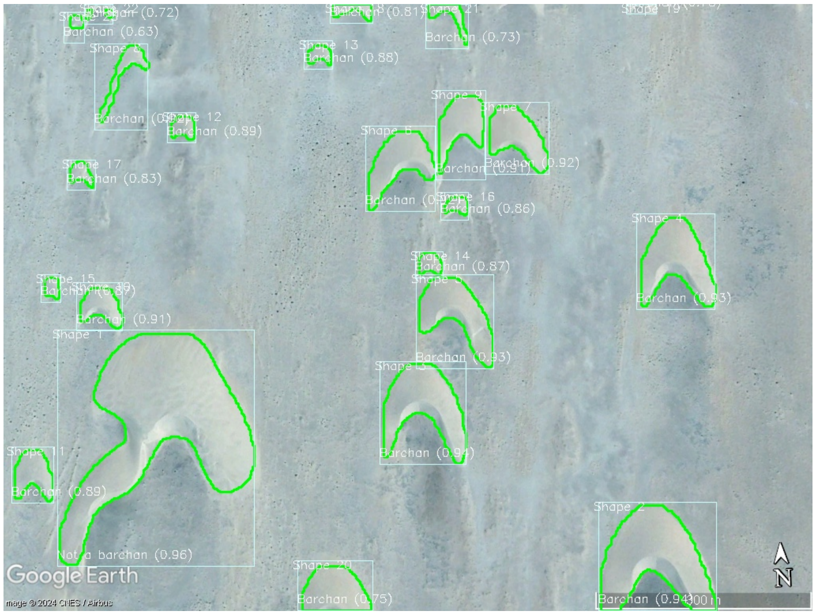

FIG. 23. Medium-resolution image (15 m per px) showing a field of barchans undergoing complex interactions on the Sahara desert: 21.314° latitude, -16.805° longitude, March 2016. Courtesy Google Earth Pro. The detection boxes, class type, confidence score, and outline are superposed with the image.

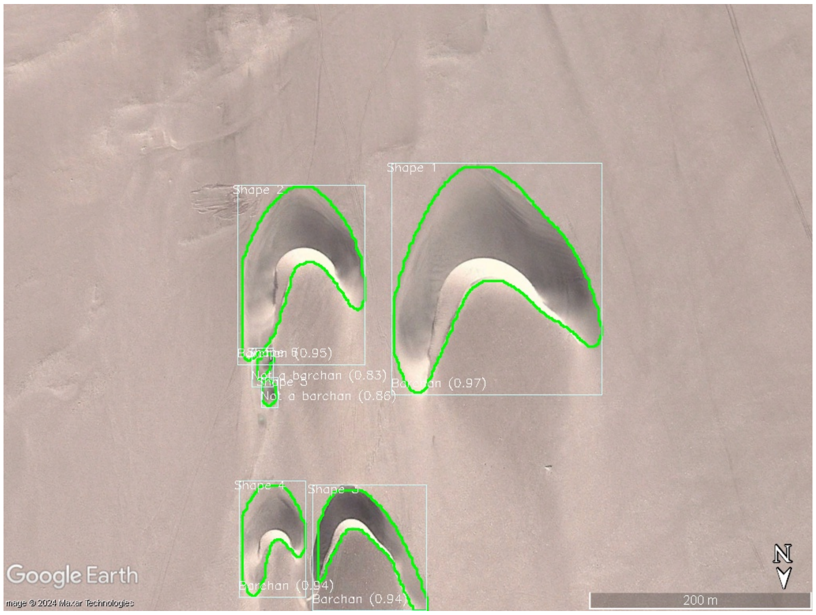

FIG. 24. Medium-resolution image (15 m per px) showing a field of barchans undergoing complex interactions on the Nazca desert: -15.278° latitude, -74.878° longitude, June 2012. Courtesy Google Earth Pro. The detection boxes, class type, confidence score, and outline are superposed with the image.

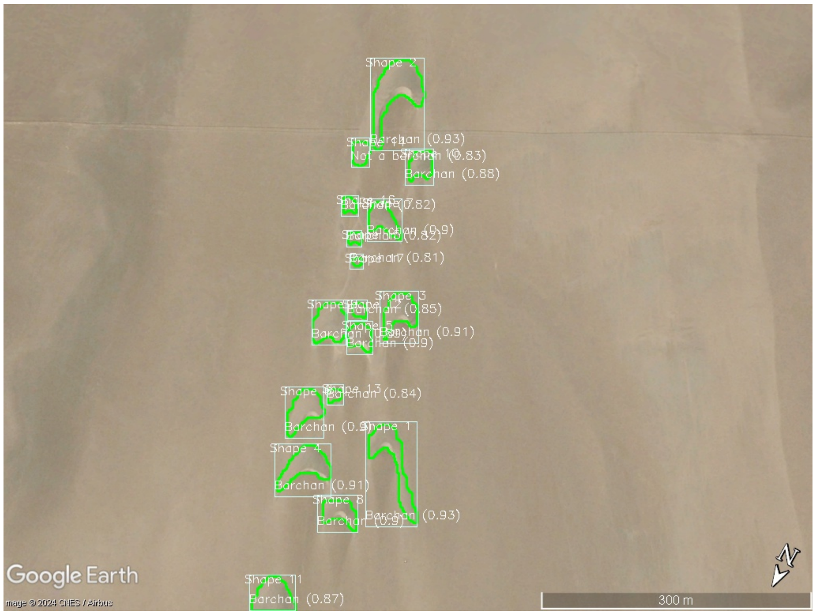

FIG. 25. Low-resolution image (30 m per px) showing a field of barchans undergoing complex interactions on the Nazca desert: -15.305° latitude, -74.875° longitude, July 2013. Courtesy Google Earth Pro. The detection boxes, class type, confidence score, and outline are superposed with the image.

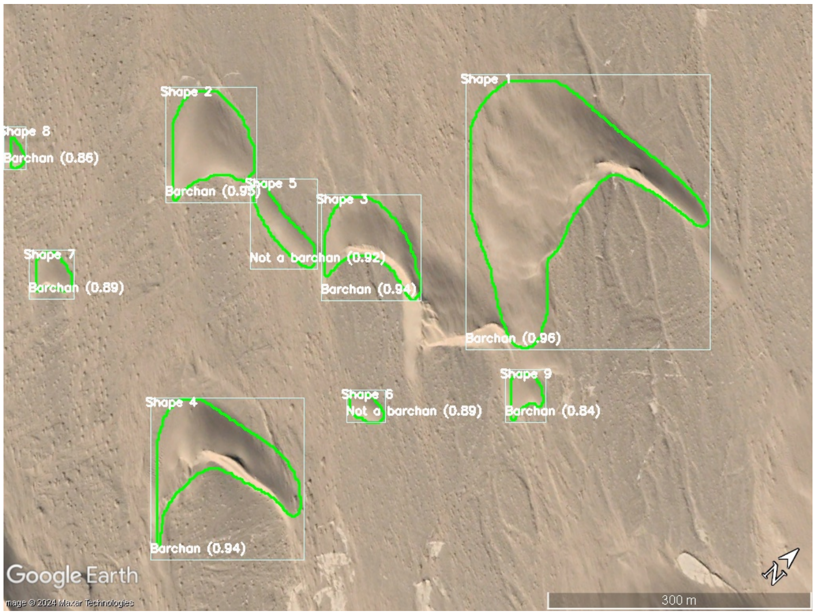

FIG. 26. Medium-resolution image (15 m per px) showing a field of barchans undergoing complex interactions on the Hexi desert: 40.012° latitude, 98.262° longitude, 2022. Courtesy Google Earth Pro. The detection boxes, class type, confidence score, and outline are superposed with the image.

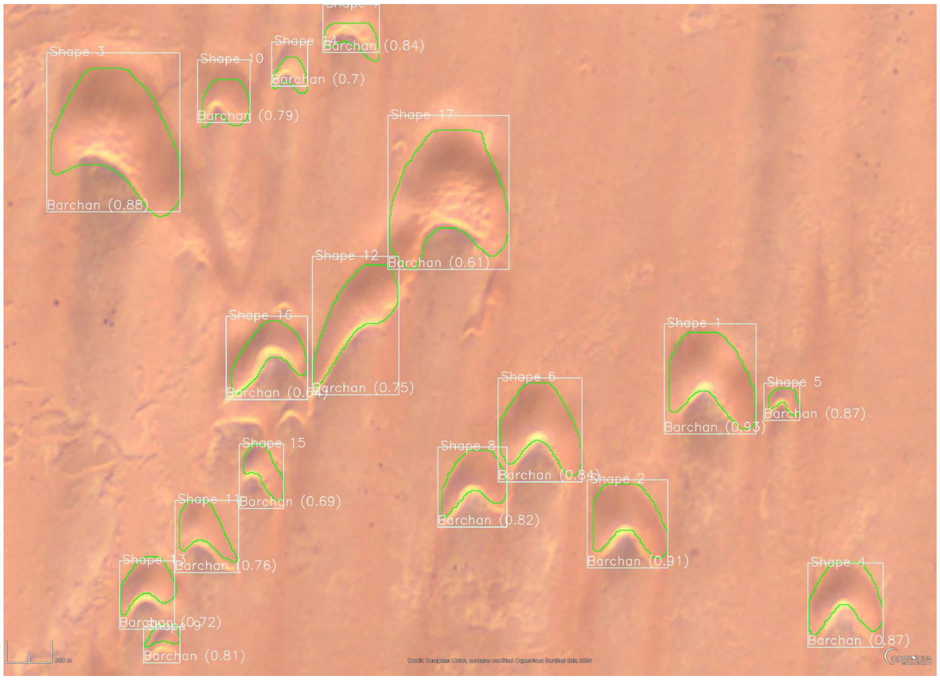

FIG. 27. First medium-resolution image (20 m per px) of a time sequence, showing a field of barchans undergoing complex interactions on the Sahara desert: 21.52° latitude, -16.73° longitude, from satellite Sentinel-2-L2A. Courtesy Copernicus EU[9]. The detection boxes, class type, confidence score, and outline are superposed with the image. Date: December 2017

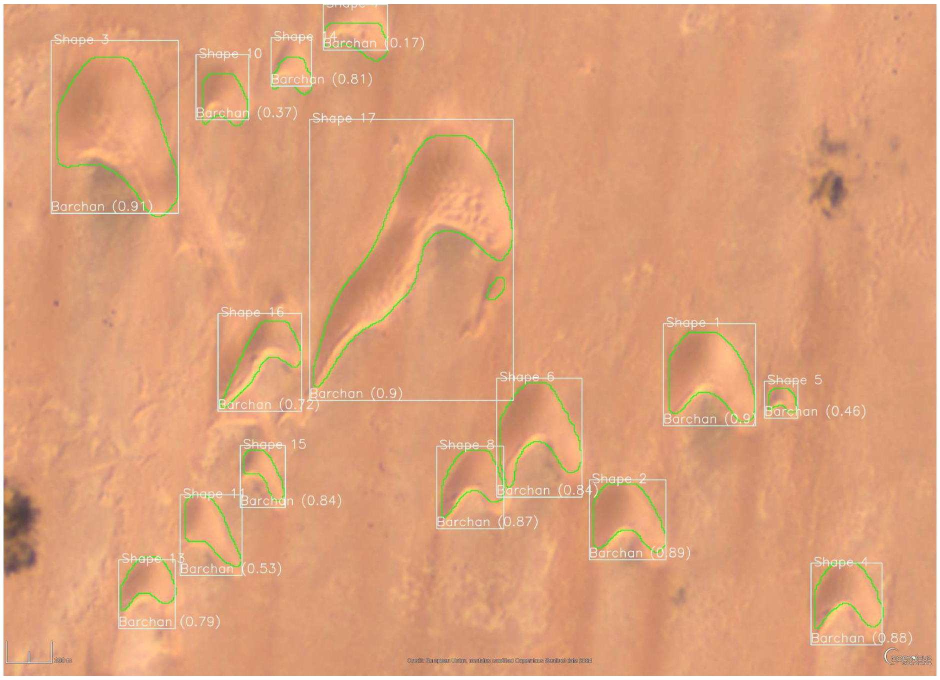

FIG. 28. Second medium-resolution image (20 m per px) of a time sequence, showing a field of barchans undergoing complex interactions on the Sahara desert: 21.52° latitude, -16.73° longitude, from satellite Sentinel-2-L2A. Courtesy Copernicus EU[9]. The detection boxes, class type, confidence score, and outline are superposed with the image. Date: February 2018

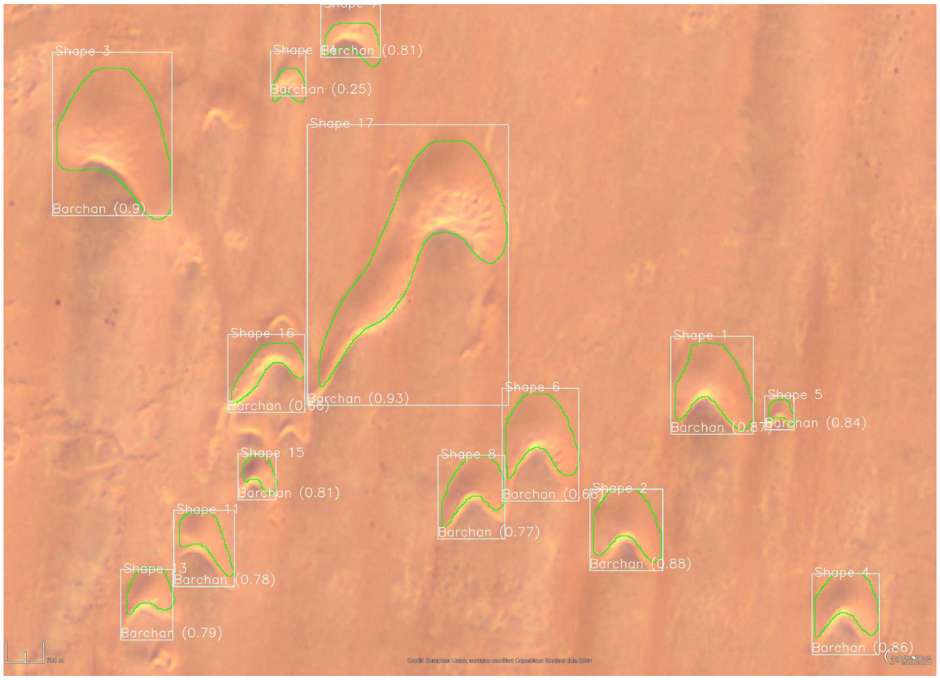

FIG. 29. Third medium-resolution image (20 m per px) of a time sequence, showing a field of barchans undergoing complex interactions on the Sahara desert: 21.52° latitude, -16.73° longitude, from satellite Sentinel-2-L2A. Courtesy Copernicus EU[9]. The detection boxes, class type, confidence score, and outline are superposed with the image. Date: January 2019

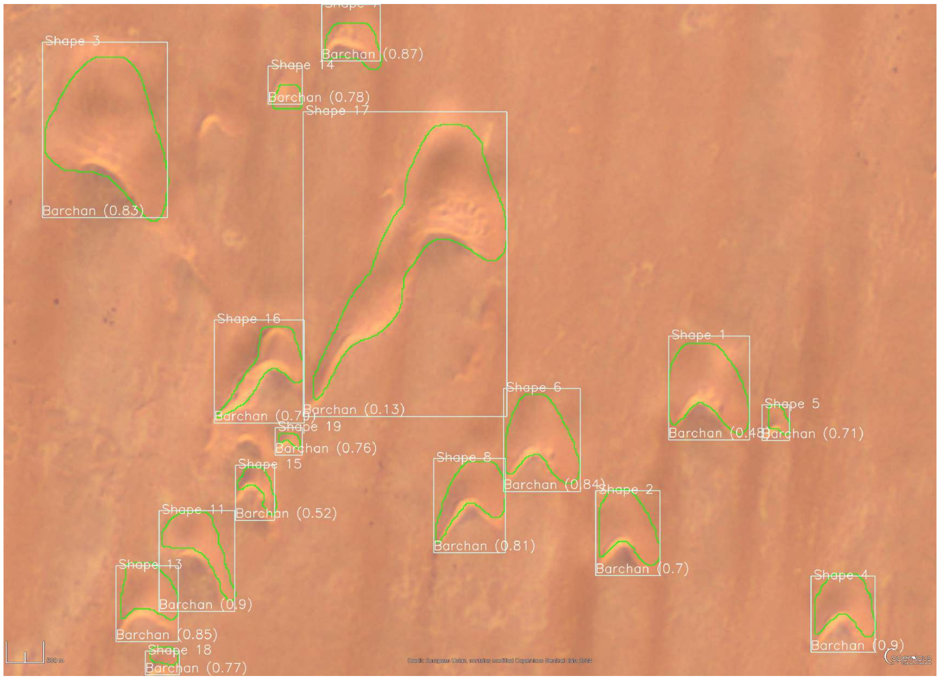

FIG. 30. Fourth medium-resolution image (20 m per px) of a time sequence, showing a field of barchans undergoing complex interactions on the Sahara desert: 21.52° latitude, -16.73° longitude, from satellite Sentinel-2-L2A. Courtesy Copernicus EU[9]. The detection boxes, class type, confidence score, and outline are superposed with the image. Date: January 2020

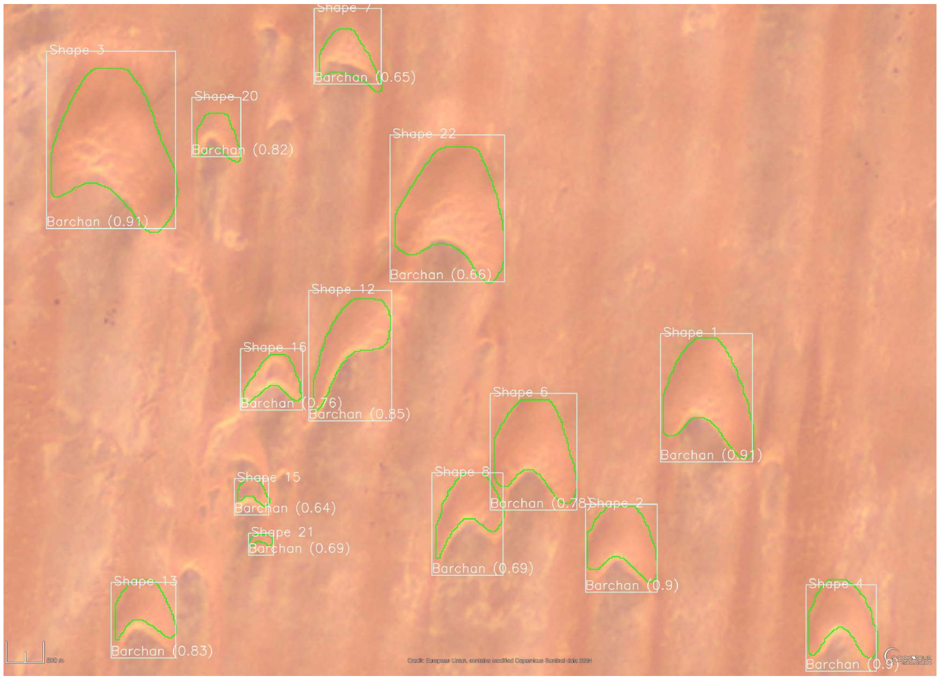

FIG. 31. Fifth medium-resolution image (20 m per px) of a time sequence, showing a field of barchans undergoing complex interactions on the Sahara desert: 21.52° latitude, -16.73° longitude, from satellite Sentinel-2-L2A. Courtesy Copernicus EU[9]. The detection boxes, class type, confidence score, and outline are superposed with the image. Date: January 2021

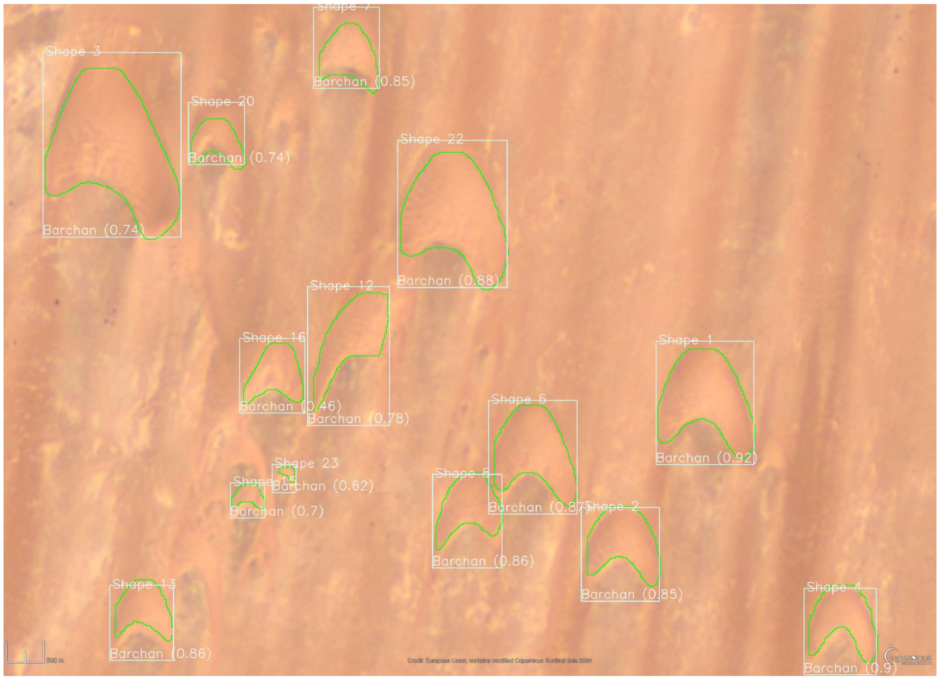

FIG. 32. Sixth medium-resolution image (20 m per px) of a time sequence, showing a field of barchans undergoing complex interactions on the Sahara desert: 21.52° latitude, -16.73° longitude, from satellite Sentinel-2-L2A. Courtesy Copernicus EU[9]. The detection boxes, class type, confidence score, and outline are superposed with the image. Date: March 2022

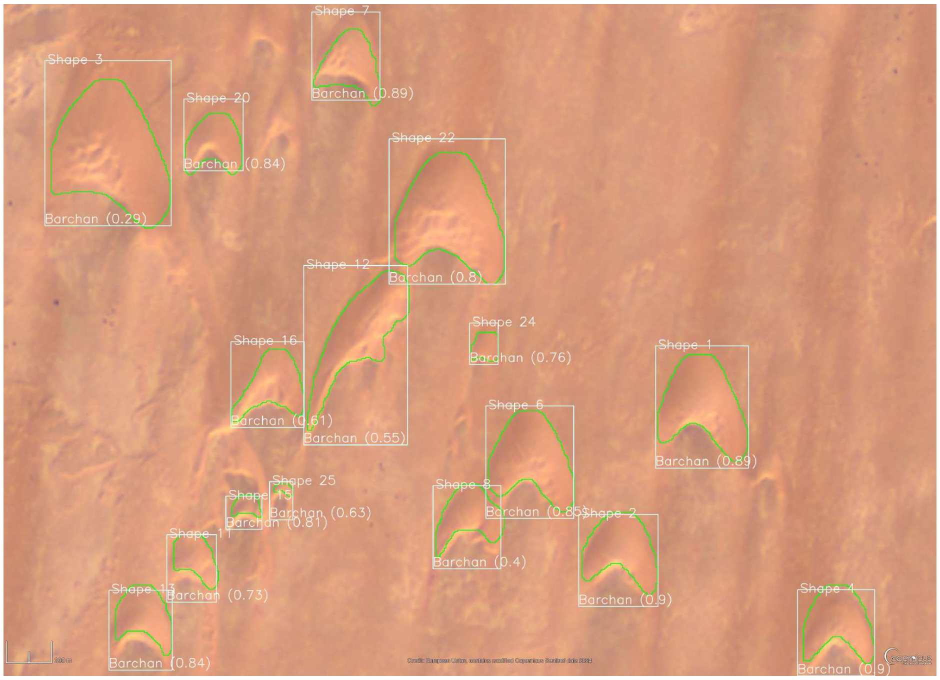

FIG. 33. Seventh medium-resolution image (20 m per px) of a time sequence, showing a field of barchans undergoing complex interactions on the Sahara desert: 21.52° latitude, -16.73° longitude, from satellite Sentinel-2-L2A. Courtesy Copernicus EU[9]. The detection boxes, class type, confidence score, and outline are superposed with the image. Date: January 2023

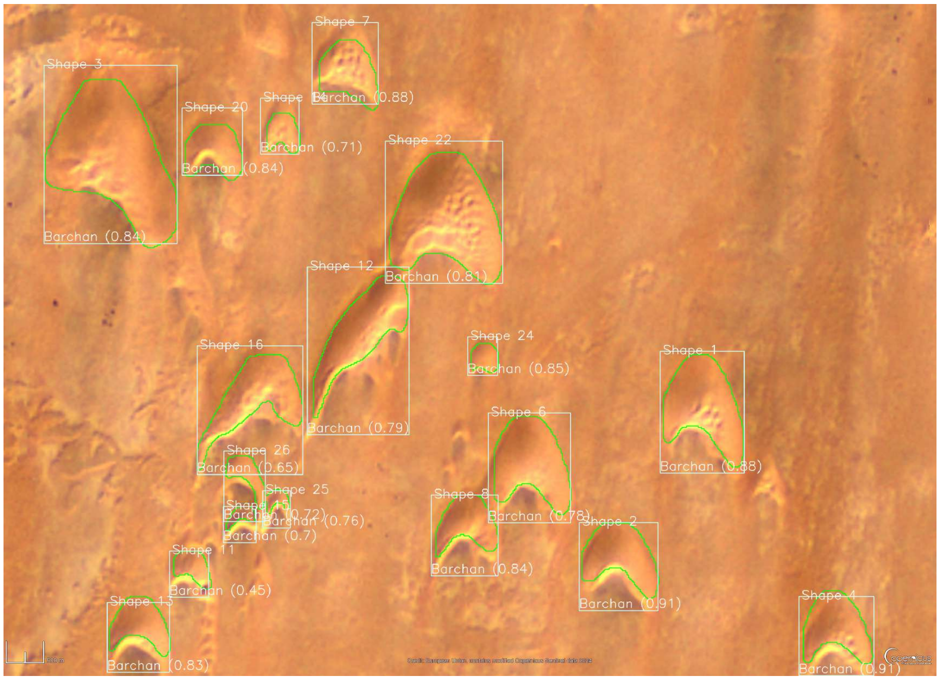

FIG. 34. Eighth medium-resolution image (20 m per px) of a time sequence, showing a field of barchans undergoing complex interactions on the Sahara desert: 21.52° latitude, -16.73° longitude, from satellite Sentinel-2-L2A. Courtesy Copernicus EU[9]. The detection boxes, class type, confidence score, and outline are superposed with the image. Date: January 2024

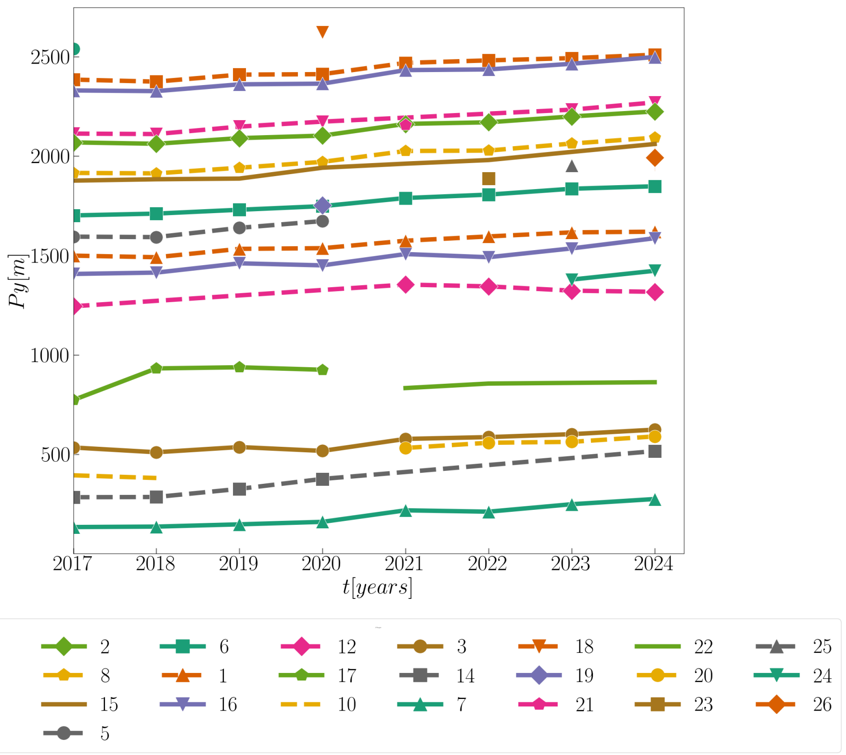

FIG. 35. Time evolution of the longitudinal position P_y of the centroid of bedforms detected in the time sequence of Figs. 27 to 34. The key shows the colors user for each tracked bedform.

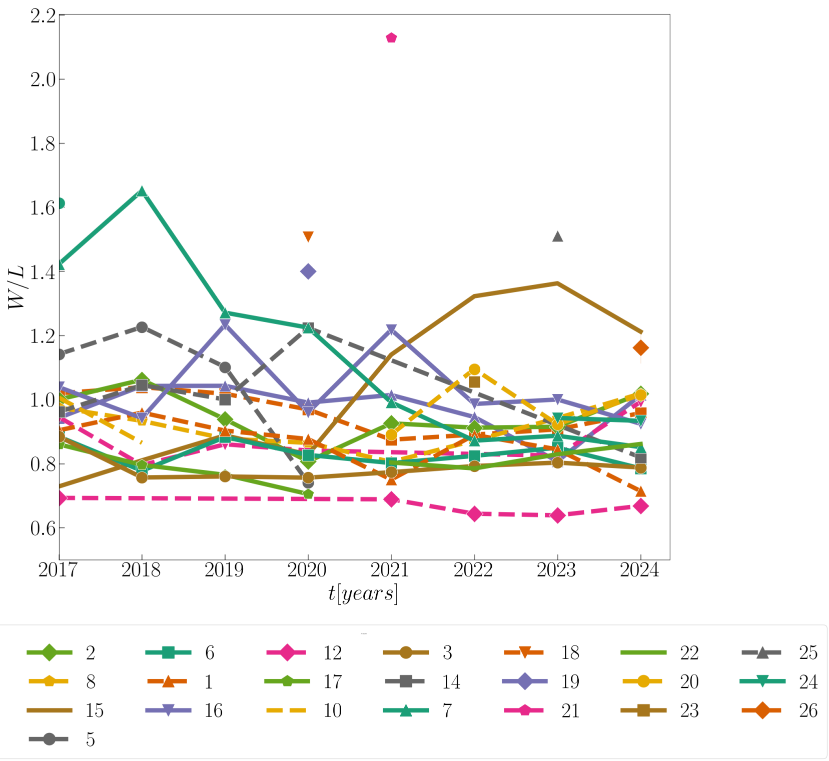

FIG. 36. Time evolution of the aspect ratio W/L of bedforms detected in the time sequence of Figs. 27 to 34. The key shows the colors user for each tracked bedform.

REFERENCES

[1]W. R. Assis and E. M. Franklin, "Experimental data on binary interactions of subaqueous barchans," *Mendeley Data* http://dx.doi.org/10.17632/jn3kt83hzh.3 (2020).

[2]W. R. Assis and E. M. Franklin, "Experimental data on barchan-barchan interaction at the grain scale," *Mendeley Data* http://doi.org/10.17632/f9p59sxm4f.1 (2021).

[3]W. R. Assis, F. Cúñez, and E. M. Franklin, "Experimental data on barchan-barchan interaction with bidisperse grains," *Mendeley Data* http://doi.org/10.17632/sbjtzbzh9k.1 (2021).

[4]E. A. Cúñez and E. M. Franklin, "Experimental dataset on "Detection and tracking of barchan dunes using artificial intelligence"," *Mendeley Data* http://doi.org/10.17632/8wh3w3y899 (2023).

[5]E. A. Cúñez and E. M. Franklin, "CNN training of experimental images for "Detection and tracking of barchan dunes using artificial intelligence"," *Mendeley Data* http://doi.org/10.17632/brgxgtpz92 (2023).

[6]E. A. Cúñez and E. M. Franklin, "CNN training of satellite images for "Detection and tracking barchan dunes using artificial intelligence"," *Mendeley Data* http://doi.org/10.17632/v4yntwdnjk (2023).

[7]W. R. Assis and E. M. Franklin, "A comprehensive picture for binary interactions of subaqueous barchans," Geophys. Res. Lett. **47**, e2020GL089464 (2020).

[8]"High resolution imaging science experiment," *HiRISE Operations Center - University of Arizona* https://www.uahirise.org/ ().

[9]"Copernicus eu," *Copernicus Browser* https://browser.dataspace.copernicus.eu/ ().
